# Biomechanical insights into the dentition of megatooth sharks (Lamniformes: Otodontidae)

**DOI:** 10.1038/s41598-020-80323-z

**Published:** 2021-01-13

**Authors:** Antonio Ballell, Humberto G. Ferrón

**Affiliations:** Shool of Earth Sciences, University of Bristol, Bristol, BS8 1RJ UK

**Keywords:** Evolution, Palaeontology

## Abstract

The evolution of gigantism in extinct otodontid sharks was paralleled by a series of drastic modifications in their dentition including widening of the crowns, loss of lateral cusplets, and acquisition of serrated cutting edges. These traits have generally been interpreted as key functional features that enabled the transition from piscivory to more energetic diets based on marine mammals, ultimately leading to the evolution of titanic body sizes in the most recent forms (including the emblematic *Otodus megalodon*). To investigate this hypothesis, we evaluate the biomechanics of the anterior, lateral, and posterior teeth of five otodontid species under different loading conditions by using two-dimensional finite element analysis. Stress distribution patterns are remarkably similar among all models under puncture and draw (i.e., when subjected to vertical and lateral forces, respectively). Contrary to expectation, higher average stress values are detected under both loading scenarios in more recent species. Altogether, this suggests little correlation between tooth morphology and key aspects of biomechanical behaviour in otodontids, making it difficult to frame the morphological trend of their dentitions within an adaptive scenario. We propose that this pattern most likely emerged as a non-functional by-product of heterochronic processes driven by selection towards larger body sizes.

## Introduction

Otodontids, colloquially referred to as megatooth sharks, constitute a family of apex predatory selachians that ranged from the Early Paleocene to the Pliocene^1–3^. This group experienced a trend towards gigantism throughout the Cenozoic that culminated with *Otodus megalodon*, the largest macropredatory shark ever to exist^4^. This species overpassed 15 m in total length and likely weighed more than 50 tons^4–7^. Historically, the evolution of such titanic body sizes in otodontids has been related to the emergence of various marine mammal lineages during the Paleogene (i.e., pinnipeds, sirenians, and cetaceans)^5,8,9^. Possessing thick layers of blubber, these taxa would have represented ideal prey for large-sized mesotherms to meet the metabolic demands of their active lifestyles^10–12^. Within this scenario, the earliest otodontids subsisted on comparatively small prey items, presumably fishes, whereas the largest and more recent species, including *O. megalodon*, consumed larger marine mammals^8,9,13,14^. This dietary shift most likely required the acquisition of a series of anatomical innovations that enabled such trophic specialisation.

The trend towards gigantism in otodontid sharks was paralleled by remarkable modifications in tooth morphology, including an increase in crown width, the loss of lateral tooth cusplets, and the acquisition of serrated cutting edges^9,14,15^. Collectively, these changes represent a shift from typical puncturing-tearing to cutting dentitions^16^. These two dental types are usually associated with different ways of capturing and processing the prey; accordingly, early otodontids were presumably adapted to prey upon small elusive animals, whereas the most recent members of this family were likely adapted to tearing flesh from large prey or carcasses^17–20^. However, only few works have assessed morphofunctional questions about shark teeth from quantitative biomechanical points of view^21–26^ and the most comprehensive studies in this regard did not find clear patterns between tooth morphology and structural resistance (i.e., the ability to withstand the effect of forces and the deformation derived from it) or puncture performance (i.e., efficiency to penetrate foodstuff). These findings called into question the classical categorization of shark dentitions into functional types^27–29^. As such, biomechanical testing of otodontid teeth is crucial for clarifying the underlying mechanisms that promoted their presumed shift in dietary preferences and better understanding the evolutionary factors that allowed them to reach the most gigantic sizes among macropredatory selachians^1,16,30^.

Here we evaluate the biomechanical behaviour of otodontid shark teeth by means of Finite Element Analysis (FEA). Borrowed from engineering, FEA is one of the most commonly used computational methods in biomechanics and functional palaeobiology^31^. This technique reconstructs the mechanical behaviour of biological structures, in terms of stress and strain, under simulated loads. To assess the functional significance of morphological trends in otodontid dentitions and test previous adaptive explanations, we analysed anterior, lateral and posterior teeth of five otodontid chronospecies (*Otodus obliquus*, *O. auriculatus*, *O. angustidens*, *O. chubutensis* and *O. megalodon*), thus capturing the diversity of dentitions exhibited by this lineage from the Palaeocene to the Pliocene^1^. We tested loading scenarios of puncture, a vertical force acting on the tip of the crown; and unidirectional draw, a lateral force acting along the distal cutting edge. Puncture was simulated under life-size absolute force estimates (i.e., bite forces that each species would have exerted in life considering their estimated body size) and scaled forces (i.e., force magnitudes scaled to maintain a constant force to surface area ratio across all models to account for morphology only^32^); and draw was simulated under scaled forces only.

## Results

**Figure 1:**
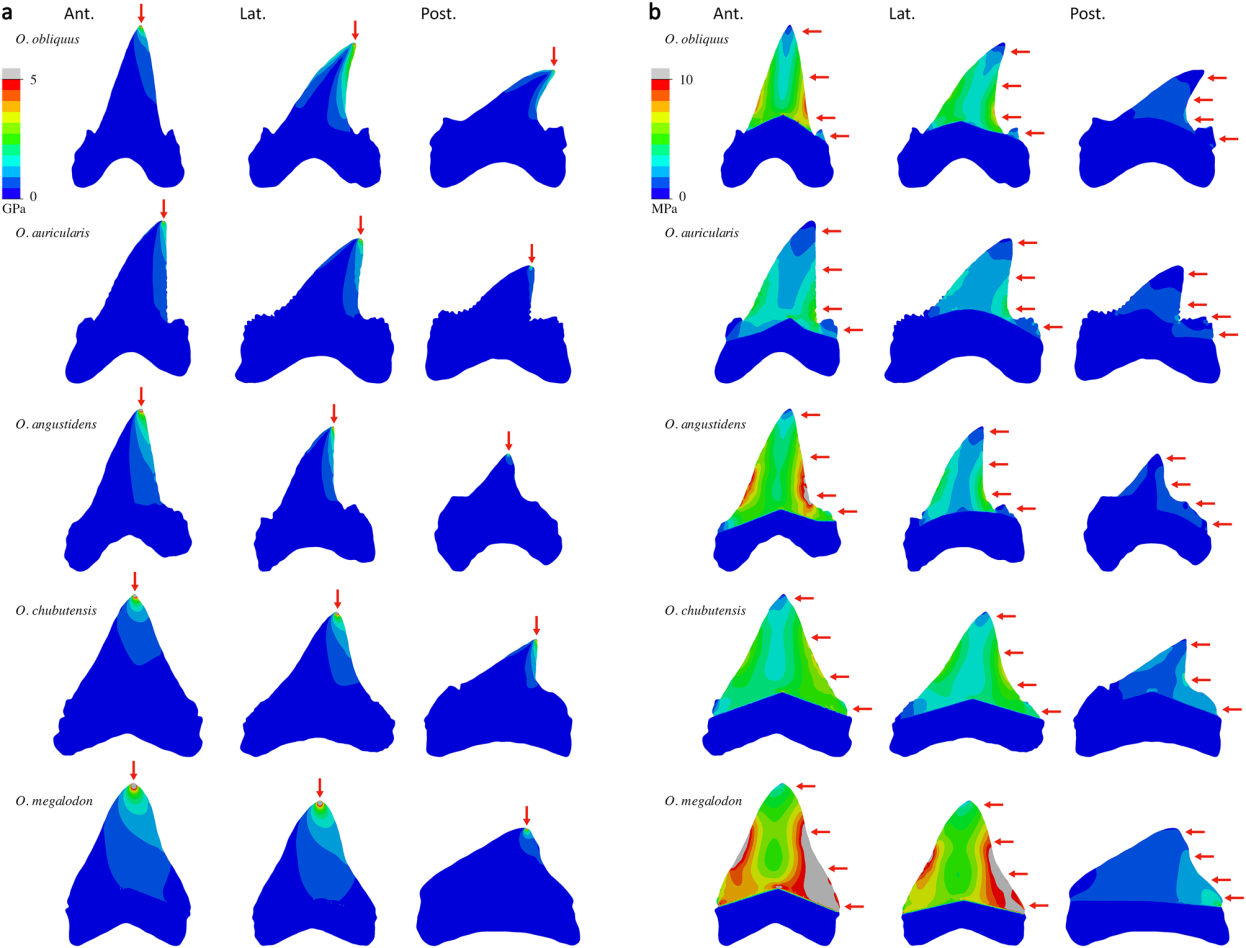
Von Mises stress distribution plots in the anterior (*Ant.*), lateral (*Lat.*), and posterior (*Post.*) teeth of the five analysed otodontid species, simulating (**a**) puncture and (**b**) draw scenarios with scaled force magnitude. Mesial is left, distal is right. Arrows indicate loading points. Grey areas represent von Mises stress values higher than 5 GPa and 10 MPa in each of the scenarios, respectively.

Finite element models under puncture scenario with scaled forces show some similarities in von Mises stress distribution patterns (Fig. 1a). The region with the highest stress is located around the tip of the main tooth crown, where the puncture force is acting, while the lowest stress is located in the root, where models are constrained. The pattern of how stress dissipates from the loading point varies among models. Stress is distributed along the center of the crown in teeth with crowns approximating an equilateral or isosceles triangular morphology, such as the anterior teeth of *O. chubutensis* and *O. megalodon*. In crowns approaching a right triangular morphology, as in the anterior teeth of *O. obliquus*, *O. auriculatus*, and *O. angustidens*, stress is mostly distributed along the distal cutting edge. Teeth with recurved crowns, as in the lateral and posterior teeth of *O. obliquus* and the lateral teeth of *O. auriculatus* and *O. angustidens*, show high stresses along the distal and (to a lesser extent) mesial cutting edges while the center of the crown exhibits low stress, a pattern resembling the bending of a typical cantilever beam (i.e., a rigid structural element supported at one end and free at the other end^33^). When puncture is simulated under estimated life-size bite force conditions for each species, same patterns of stress distribution are obtained but stress values are higher due to the higher force magnitudes that are applied (Supplementary Fig. S1). Comparing different tooth positions for the same taxa in this simulation, lateral and especially posterior teeth experience higher stresses than anterior teeth as a result of (1) experiencing higher forces because jaws behave as third-class levers, in which output (bite) forces increase towards the jaw joint; and (2) having less surface area due to their smaller size (see “Methods” section).

In the draw scenario with scaled force loadings, all models exhibit similar general distributions of stress (Fig. 1b). The portions of the teeth exhibiting the lowest stresses are the root and the very apex of the crown. The highest stress values are located along the distal cutting edge of the main crown, where the draw load is acting, as well as along the mesial cutting edge, in resemblance to a cantilever-bending scenario. The centre of the tooth crown between the cutting edges exhibits relatively lower stress, akin to the neutral axis of the beam. In taxa with lateral cusplets, namely the four oldest species, these structures show moderate to low stresses, generally higher in the distal cusplet than in the mesial, where the draw load is not acting directly.

**Figure 2:**
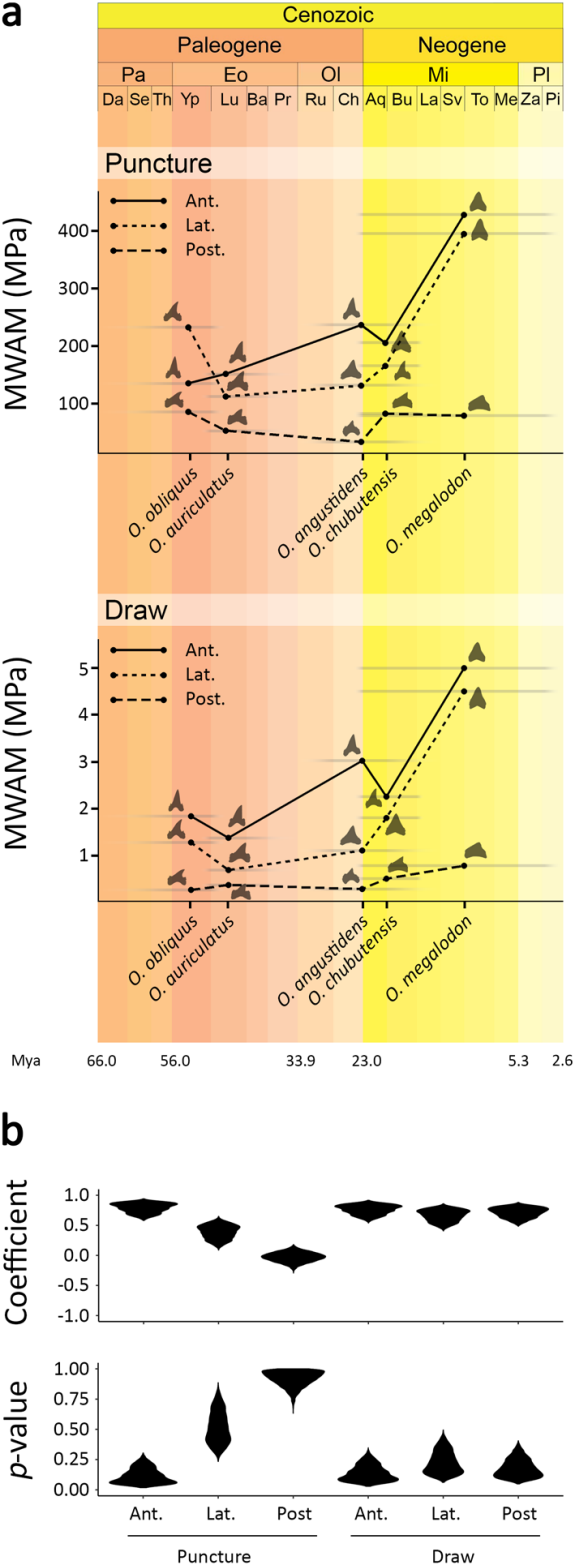
(**a**) Von Mises stress mesh-weighted arithmetic means (MWAM) calculated for anterior (*Ant.*), lateral (*Lat.*), and posterior (*Post.*) teeth of the five analysed otodontid species, simulating puncture and draw scenarios with scaled force magnitude. Data are shown in a temporal context (in million years ago, *Mya*) where stratigraphic range of each taxa is represented by grey bars (stratigraphic ranges based on Cappetta^1^ and Diedrich^9^). (**b**) Density distributions of coefficients and *p* values derived from correlation analyses between von Mises stress MWAM and species age (randomly selected within their chronostratigraphic range, n = 10,000). Epoch: Pa, Paleocene; Eo, Eocene; Ol, Oligocene; Mi, Miocene; Pl, Pliocene; Age: Da, Danian; Se, Selandian; Th, Thanetian; Yp, Ypresian; Lu, Lutetian; Ba, Bartonian; Pr, Priabonian; Ru, Rupelian; Ch, Chattian; Aq, Aquitanian; Bu, Burdigalian; La, Langhian; Sv, Serravalian; To, Tortonian; Me, Messinian; Za, Zanclean; Pi, Piacenzian.

Some general patterns can be extracted from comparing the von Mises stress mesh-weighted arithmetic means (MWAM) across finite element models (Fig. 2). Under both puncture and draw scaled force loadings, relative stresses decrease as tooth position becomes more distal, with the exception of the anterior and lateral teeth of *O. obliquus* during puncture (Fig. 2a). When comparing different species, the teeth of older species display lower stresses under both loading regimes than those of more recent taxa, although there are some exceptions (Fig. 2a). For example, *O. obliquus* shows higher stress than *O. auriculatus* when comparing lateral and posterior teeth during puncture, and anterior and lateral teeth during draw. Similarly, the anterior teeth of *O. angustidens* exhibit higher stress than those of the younger species *O. chubutensis*, during both puncture and draw. In general, the greatest differences in stress magnitude among taxa are seen in anterior and lateral teeth, while posterior teeth show more similar stress values under both loading conditions. Correlation analyses support a general trend towards increasing von Mises stress MWAM in time for all tooth positions and loading scenarios, apart from posterior teeth under puncture where no trend is detected (Fig. 2b). These patterns remain broadly consistent when von Mises stress MWAM is calculated considering only elements of the crown tooth, with the exception of the teeth of *O. obliquus* which show higher stress values during both puncture and, to a lesser extent, draw (Supplementary Fig. S2).

## Discussion

Otodontid teeth show general patterns of stress distribution similar to those of extant elasmobranchs^29^, with high stresses concentrated around the crown apex and along the mesial and distal cutting edges during puncture and draw, respectively (Fig. 1). FE models do not reveal structural weaknesses that could potentially lead to failure under both loading scenarios in any of the considered teeth despite the high force magnitudes that were simulated (Fig. 1 and Supplementary Fig. S1). Stress patterns during draw are consistent with cantilever beam bending^33^, especially in anterior and lateral teeth that exhibit higher and straighter crowns (Fig. 1b), and similar to those of extant species with elongate tooth crowns^29^. As such, despite covering a relatively diverse range of shapes, with typical examples of distinct dental types^1,9,15^, the teeth of different otodontid species exhibit similar patterns of stress distribution in both puncture and draw. This suggests that dental morphology is not a reliable proxy for functional performance^18,27–29^, which undermines the traditional categorization of shark teeth into morphofunctional classes (i.e., specific dental morphotypes presumably adapted to clutching, tearing, cutting, crushing, or grinding) employed for decades to support dietary and ecological interpretations in both living and extinct groups^1,16,30^.

Von Mises stress mesh-weighted arithmetic mean (MWAM) decreases towards more distal tooth positions within otodontid species (Fig. 2a), indicating that the robust, shorter crowned posterior teeth are structurally more resistant than the gracile anterior and lateral teeth. This suggests that heterodonty in the dentition of otodontids could be a response to mechanical constraints where the morphology of more distal teeth is determined, at least in part, by the need to resist higher bite forces. When comparing different taxa, the teeth of older species exhibit, with few exceptions, lower von Mises stress values than those of more recent ones during both puncture and draw (Fig. 2). However, this trend is not consistent with the mechanical properties presumed a priori for the dental types found in taxa possessing extreme dental morphologies. Extant sharks with puncturing-tearing dentitions, similar to that of *O. obliquus*, usually pierce and hold soft prey between their jaws before swallowing them with little manipulation^34^; in contrast, species with cutting dentitions, similar to that of *O. megalodon*, slice off large pieces of flesh through a combination of vertical bites and lateral head shaking^35,36^. Fossil evidence supports that the latest otodontids (i.e., *O chubutensis* and *O. megalodon*) also possessed the ability to bite and crush the bones of pinnipeds, sirenians, and cetaceans during hunting or scavenging^8,13,37–40^. This implies that the teeth of these species would impact hard mineralized endoskeletal tissues more often than those of their earlier relatives that are presumed to have fed mostly on fish^9,41^. From this perspective, an optimization in draw and, probably, puncture performances is expected through the evolution of otodontid dentitions in order to support higher loads; an expectation not substantiated by our results. In any case, the evaluation of these aspects should be conducted with caution given the complexity of feeding kinematics in sharks^17–20^ and the potential effects of interspecific variation in the labio-lingual thickness and histology of the teeth. Nonetheless, planar (two-dimensional) models have been established as a useful alternative to three-dimensional models for capturing reaction forces and comparative patterns of stress and strain^42^. Our data support this approach given the remarkable similarities between the general patterns of stress distribution recovered here for otodontid teeth (Fig. 2), based on planar models with homogeneous material properties, and those previously reported for a number of living sharks, based on more complex three dimensional models accounting for both the distribution and properties of the different dental tissues^29^.

Our results reveal that the morphological trend recorded in otodontid dentitions is difficult to frame within a functional context^9,14,15^, thus calling into question its adaptive significance during the dietary transition of this group and, ultimately, its causal impact on the evolution of gigantic body sizes in the most derived species. The presence of serrated edges (not captured in our FE models) is usually considered as a character related to increased cutting efficiency^18,22,30^. Accordingly, the evolution of serrations in the tooth cutting edges of both otodontids and the great white shark (i.e., *Carcharodon carcharias*)^43–45^ are interpreted as independent adaptations to improve cutting performance triggered by the acquisition of comparable diets based mostly on marine mammals^9,46^. However, the functional role of this feature has been challenged by recent biomechanical studies on shark teeth^28^ and the question of whether the acquisition of edge serrations in otodontids had some impact on their ability to prey upon marine mammals^9^ will remain unanswered until dynamic testing is conducted specifically on these taxa^26^. Biomechanical testing of the cutting mechanics and efficiency of complete tooth rows could also provide relevant functional insights in this context by revealing emergent functional properties of the dentition as a whole. In analogy with the extant great white shark^46^, the dentitions of more recent species of otodontids might have comprised a continuous cutting edge spanning from one commissure through the symphysis to the opposed commissure and provided with two orders of serrations (i.e., the teeth and the serrae sensu stricto). Besides potential anatomical specializations, the dietary shift that occurred within Otodontidae may have been an intrinsic consequence of body size increase^4^, allowing them to consume larger prey^47,48^, facilitated by the pre-existence of highly active metabolisms and mesothermy in smaller preceding forms^10,11^.

**Figure 3:**
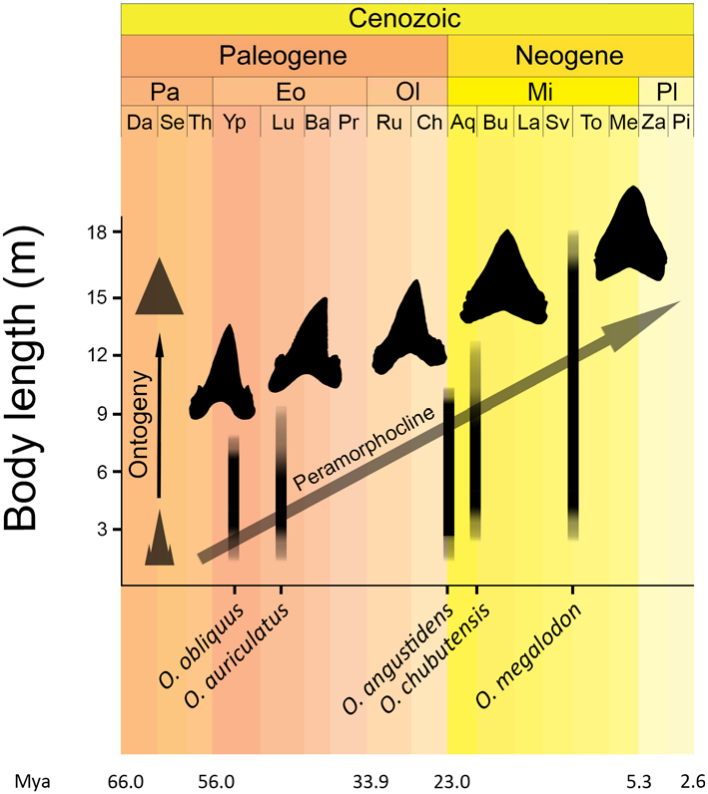
Schematic representation of the trends in tooth morphology, body size and presumed heterochronic phenomena through the evolution of otodontid sharks.

In the absence of convincing functional evidence, other non-adaptive processes should be considered when attempting to explain the morphological changes in the dentition of otodontids. Body size selection triggered by heterochrony (i.e., changes to the timing or rate of developmental events, relative to an ancestor^49^) can produce trends in traits that exhibit allometric variation (i.e., changes in morphology associated with size variation)^50^. Heterochrony had a relevant role in the evolution of gigantism in otodontids^51^, where a general trend towards the expansion of the somatic growth (i.e., peramorphosis) is recorded in the vertebral rings of successive taxa^14^. This phenomenon appears to be a product of an increased rate of growth (i.e., acceleration) and a delayed offset timing (i.e., hypermorphosis) in more recent species^14^. These heterochronic changes are mirrored in the dentitions of otodontids and are fundamental for understanding the ontogenetic and interspecific variation of tooth morphology within the group^52–54^. When considered in the context of heterochrony, the evolution of otodontid dentitions can be framed within a continuous morphological gradient where progressively larger and more peramorphic species pass through more developmental stages during ontogeny, a trend that can be expressed as a peramorphocline^55,56^ (Fig. 3). This may explain why the ontogenetic change in *O. megalodon* teeth mimics the modifications that took place during their evolution within Otodontidae^53,54,57,58^. We propose that the morphological differences among otodontid dentitions may not be the result of selection acting on those traits but are simple sequelae of size variation. Interestingly, a similar pattern is present within lamnid sharks (i.e., *Carcharodon*, *Isurus* and *Lamna* genera and extinct relatives), where size and similar aspects of tooth morphology (i.e., crown width and presence/absence of lateral cusplets) covary in comparable ways both along their ontogeny^59–62^ and throughout phylogeny^44,63^. In fact, heterochronic processes may have also been fundamental in the shaping of dental morphological diversity of extinct and living lamnid species^63^. Disentangling the causes that underly these phenomena in different groups, and ascertaining whether they respond to common functional demands and/or developmental mechanisms, might inform about the homology of key characters in these groups (e.g., lateral cusplets)^60^ and ultimately could provide new insights into their debated affinities^5,8,9,43–45,53^ by guiding character selection in future phylogenetic analyses. From this perspective, and in agreement with the biomechanical evidence presented here, the long-term changes in the general morphology of otodontid teeth might be better considered as a non-functional by-product of heterochronic phenomena, most likely driven by selection on life history traits favouring the attainment of larger body sizes.

## Methods

### Model creation

Images of teeth in labial view were obtained for the otodontid species *Otodus obliquus*, *O. auriculatus*, *O. angustidens*, *O. chubutensis* and *O. megalodon* from the literature and from specimens in museum collections (Supplementary Table S1). We follow the taxonomic nomenclature of Cappetta^1^ and refer to that study for a detailed discussion on alternative existing nomenclatures. For each species, we considered teeth from the upper jaw with anterior, lateral and posterior positions in order to span the morphological diversity of otodontid teeth related to heterodonty (i.e., anterior I–III, lateral III–IV, and posterior II–III, following the terminology of Applegate & Espinosa-Arrubarrena^53^ and Diedrich^9^; see Supplementary Fig. S3). The images were imported into ImageJ v. 1.51r^64^ and the outline of each tooth was drawn using the multipoint tool. The XY coordinates of the outline were obtained using Microsoft Excel and imported into the CAD software Inventor Professional 2016 (Autodesk). The outline was sketched from the XY coordinates and the planar models were exported as STP files (available at the Open Science platform Figshare, https://figshare.com/s/e245548d6f31b226a7b0).

### Bite force estimations

Anterior and posterior vertical bite forces were estimated for each species under the assumption that bite force increases at 0.67 the power of body mass^65^. Estimations were made presuming isometry from values obtained in a jaw model of a 240 kg great white shark specimen (i.e., anterior and posterior bite forces of 1602 N and 3131 N, respectively)^66^. The arithmetic average of anterior and posterior force values was considered as the force exerted by the lateral region of the jaw. The body mass of each species was estimated from exponential models established in living great white sharks^5^ using body length estimates reported in the literature (Supplementary Table S1).

### Finite element analysis

Two-dimensional FEA was performed in Abaqus v. 6.14-1 (Simulia). Tooth planar models were meshed prior to the analyses, using three-node linear triangular elements of type CPE3. The optimal number of elements was determined in a convergence test, using the *O. megalodon* lateral tooth model as a reference (Supplementary Fig. S4). Different element sizes were chosen for meshing in order to maintain similar numbers of finite elements across models (from 29,323 to 42,922) (Supplementary Table S2).

Tooth models were assigned the elastic, isotropic, and homogeneous material properties of lamniform osteodentine, with Young’s modulus of 28.44 GPa^67^ and Poisson’s ratio of 0.3^68^. Enameloid was not modelled as the distribution and thickness of this tissue is virtually unknown for most otodontids^69^ and osteodentine represents the vast majority of the tooth volume in lamniform sharks^70^. Boundary conditions were applied by constraining all nodes within the tooth root in all three degrees of freedom (U_1_, U_2_ and U_R1_). The loose attachment of teeth to the dental ligament of shark jaws allows some degree of movement, especially in the labiolingual direction. However, the mechanics of these movements are poorly understood and thus difficult to simulate^23,24,29^. Additionally, our planar models do not capture the labiolingual axis, which is the main direction of tooth oscillation. Thus, we assumed our models to be static in translation and rotation along the apicobasal and mesiodistal axes, following previous approaches^29^.

FEA was performed under two loading scenarios: (1) puncture, simulating a vertical bite force applied to the apex of the tooth crown; and (2) unidirectional draw, simulating a horizontal lateral force applied along the distal cutting edge of the tooth crown. In the puncture simulation, the force was applied to a single node and two sets of analyses were performed. The first one used bite forces taking into account size (see above), providing an estimate of the different absolute bite forces that each species would have experienced in different tooth positions (Supplementary Table S3). A second analysis with scaled force magnitudes was performed to remove the effect of size and compare shape differences only. The bite forces were scaled according to model surface area (Supplementary Table S3), using the *O. megalodon* anterior tooth model (49,051 N) as a reference, so as to keep the same F/SA ratio and allow shape comparisons^32^. The draw load was applied to a set of nodes defining the mesial edge of the tooth crowns. An arbitrary magnitude of 500 N, following previous works^29^, was used for the *O. megalodon* anterior tooth model, and this force was scaled in the rest of the models to keep the same F/SA ratio and account for shape only. The total draw force magnitude was divided by the number of nodes to which the force was applied (Supplementary Table S3).

FEA results were summarised in field outputs including von Mises stress, a commonly used parameter in palaeobiology^71^ which predicts failure under ductile fracture^32,72^. Areas of the models showing high stress values indicate points of structural weakness which are more susceptible to failure. The von Mises stress mesh-weighted arithmetic mean (MWAM) was calculated to account for element size differences within non-uniform meshes^73^ considering finite elements from both the whole tooth and the tooth crown. Temporal trends in von Mises stress MWAM were assessed with Pearson correlation analyses. Correlation between MWAM and species age was evaluated accounting for the uncertainty associated to the duration of each taxon. For this purpose, repeated correlation analyses (n = 10,000) were performed, where species ages were randomly subsampled within their respective chronostratigraphic ranges. Derived correlation coefficients and *p*-values were displayed as violin density plots generated using the package ‘ggplot2’^74^. All the analyses were performed in in R^75^ and resulting scripts are available at the Open Science platform Figshare (https://figshare.com/s/e245548d6f31b226a7b0).

## Supplementary Information

Supplementary Information

## Data Availability

The data set as well as the R syntax used for the analyses presented here are available at the Open Science platform Figshare (https://figshare.com/s/e245548d6f31b226a7b0).
