# Supplementary material for: Biomechanical insights into the dentition of megatooth sharks (Lamniformes: Otodontidae)
Source: Sci Rep. 2021 Jan 13;11:1232. doi: 10.1038/s41598-020-80323-z (PMC7806677; doi:10.1038/s41598-020-80323-z)
Supplement: Supplementary file 1 — Supplementary Information [file 41598_2020_80323_MOESM1_ESM.pdf]

Supplementary information

Biomechanical insights into the dentition of megatooth sharks (Lamniformes: Otodontidae)

Antonio Ballell[1], and Humberto G. Ferrón[1,*]

[1]Shool of Earth Sciences, University of Bristol, Bristol BS8 1RJ, UK.

*humberto.ferron@bristol.ac.uk

This file includes:

Supplementary Figure 1. Von Mises stress distribution plots in the anterior (*Ant.*), lateral (*Lat.*), and posterior (*Post.*) teeth of the five analysed otodontid species, simulating puncture scenario with life-size absolute force estimates.

Supplementary Figure 2. Mesh-weighted average von Mises stress (MWAM) values calculated for anterior, lateral, and posterior teeth of the five analysed otodontid species, simulating puncture and draw scenarios with scaled force magnitude. This parameter was determined considering finite elements in the crown tooth.

Supplementary Figure 3. Tooth sets of the different otodontid species considered in our study.

Supplementary Figure 4. Convergence test results showing the variation of the mesh-weighted average von Mises (MWAM) stress respect to element size.

Supplementary Table 1. Details of planar models, tooth height, and body dimensions considered in the present study.

Supplementary Table 2. Summary of mesh parameters.

Supplementary Table 3. Finite element model characteristics and loading scenarios. *F* force, *SA* surface area.

Supplementary References

Other supplementary materials include (available at the Open Science platform Figshare):

Supplementary Data 1. STP files of planar models.

Supplementary Data 2. R code and associated files.

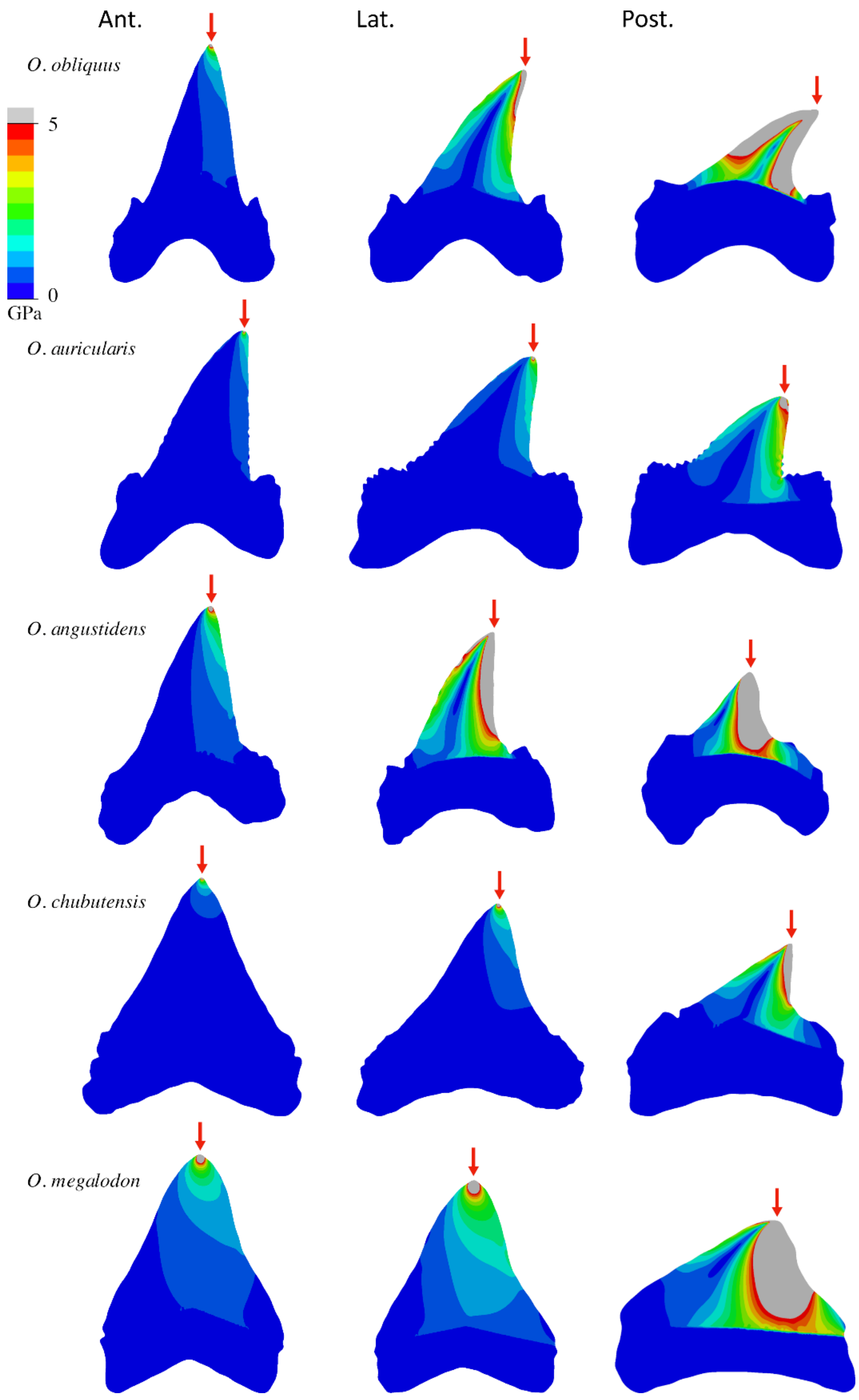

Supplementary Figure 1. Von Mises stress distribution plots in the anterior (*Ant.*), lateral (*Lat.*), and posterior (*Post.*) teeth of the five analysed otodontid species, simulating puncture scenario with life-size absolute force estimates. Mesial is left, distal is right. Arrows indicate loading points. Grey areas represent von Mises stress values higher than 5 GPa.

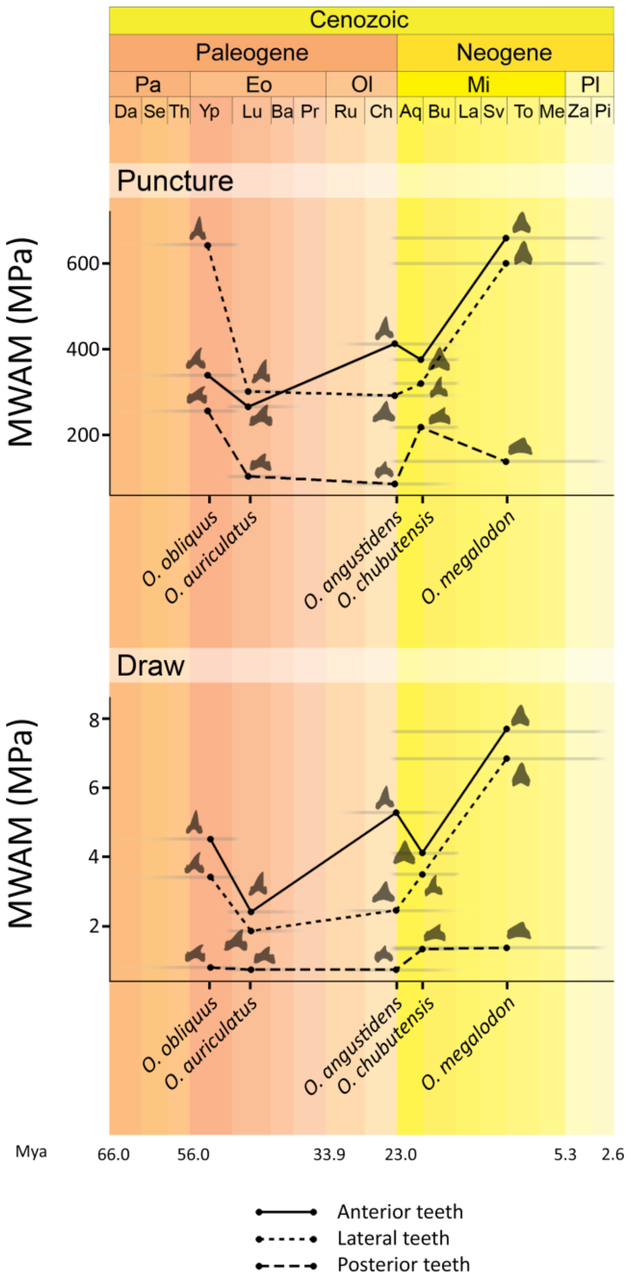

Supplementary Figure 2. Mesh-weighted average von Mises stress (MWAM) values calculated for anterior, lateral, and posterior teeth of the five analysed otodontid species, simulating puncture and draw scenarios with scaled force magnitude. This parameter was determined considering finite elements in the crown tooth. Data are shown in a temporal context (in million years ago, *Mya*) where stratigraphic range of each taxa is represented by grey bars (stratigraphic ranges based on Cappetta[1] and Diedrich[2]). Epoch: Pa, Paleocene; Eo, Eocene; Ol, Oligocene; Mi, Miocene; Pl, Pliocene; Age: Da, Danian; Se, Selandian; Th, Thanetian; Yp, Ypresian; Lu, Lutetian; Ba, Bartonian; Pr, Priabonian; Ru, Rupelian; Ch, Chattian; Aq, Aquitanian; Bu, Burdigalian; La, Langhian; Sv, Serravalian; To, Tortonian; Me, Messinian; Za, Zanclean; Pi, Piacenzian.

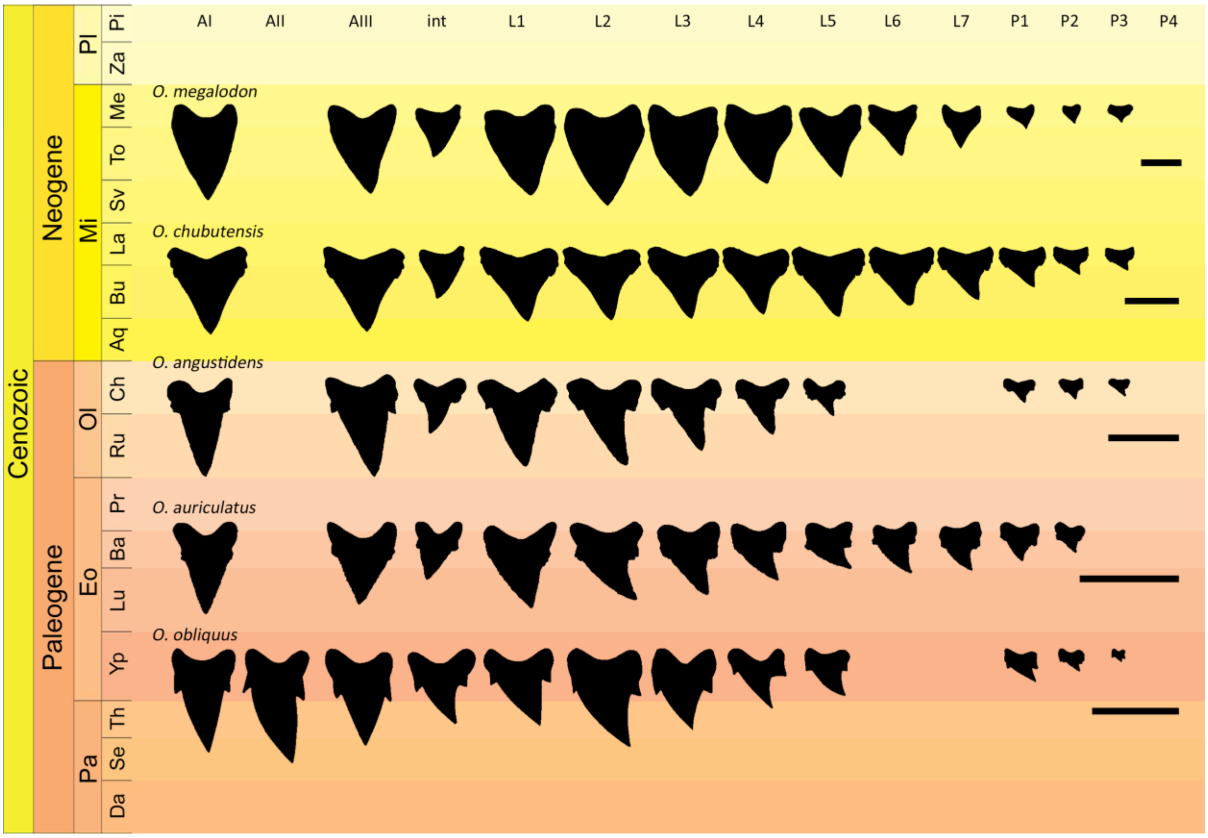

Supplementary Figure 3. Tooth sets of the different otodontid species considered in our study illustrating the morphology of anterior (A), lateral (L), intermediate (int) and posterior (P) teeth. Otodontid dentitions show a clear monognathic heterodonty (i.e., the morphology of their teeth varies depending on their position along the jaw). Thus, crowns of anterior teeth (situated close to the symphysis, left) are gracile and elongated while those of posterior teeth (situated close the commissures, right) are shorter and more robust. Lateral teeth show intermediate morphologies. Tooth set drawings modified from Perez et al.[3] and Applegate & Espinosa-Arrubarrena[4]. Epoch: Pa, Paleocene; Eo, Eocene; Ol, Oligocene; Mi, Miocene; Pl, Pliocene; Age: Da, Danian; Se, Selandian; Th, Thanetian; Yp, Ypresian; Lu, Lutetian; Ba, Bartonian; Pr, Priabonian; Ru, Rupelian; Ch, Chattian; Aq, Aquitanian; Bu, Burdigalian; La, Langhian; Sv, Serravalian; To, Tortonian; Me, Messinian; Za, Zanclean; Pi, Piacenzian. Scale bars equal 5 cm.

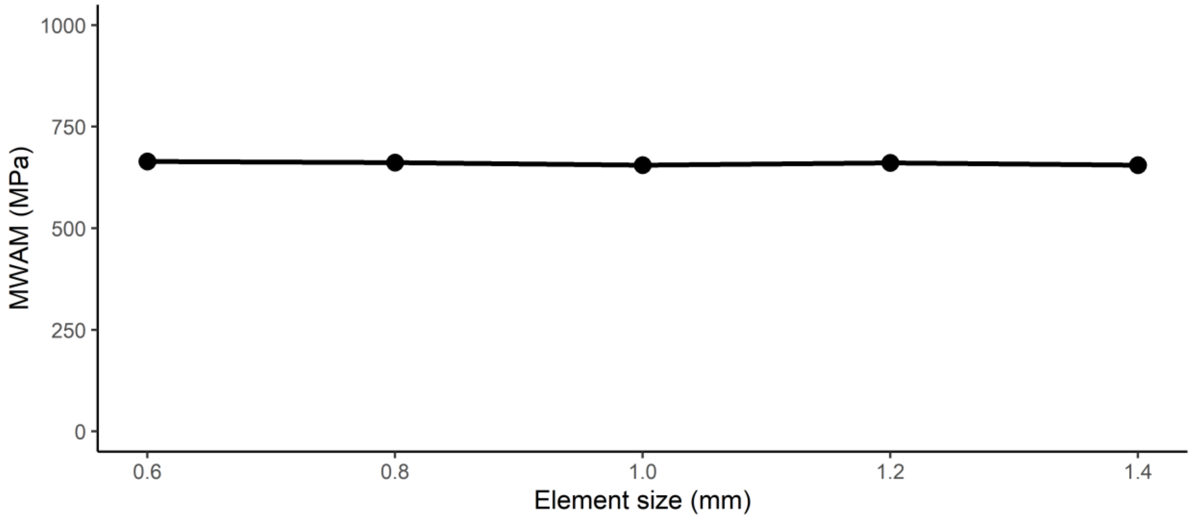

Supplementary Figure 4. Convergence test results showing the variation of the mesh-weighted average von Mises (MWAM) stress respect to element size. Percentage error below 1 in all cases (see Supplementary Data S2).

	Tooth	Planar model reference	Tooth heigth (cm)	Tooth height source	Body length (m)	Body length reference	Body mass (kg)
O. obliquus	A	Gordon Hubbell collection (ds1001 UA1)	6.7	Gordon Hubbell collection natural tooth set	600	Biton-Porsmoguer[5]	2162.96
	L	Gordon Hubbell collection (ds1001 UL2)	5.4	Gordon Hubbell collection natural tooth set	600	Biton-Porsmoguer[5]	2162.96
	P	Gordon Hubbell collection (ds1001 UL10)	1.7	Gordon Hubbell collection natural tooth set	600	Biton-Porsmoguer[5]	2162.96
O. auriculatus	A	Diedrich[2]: fig. 3: 1	4.7	Applegate and Espinosa-Arrubarrena[4]	400	Applegate and Espinosa-Arrubarrena[4]	597.22
	L	Cappetta[1]: fig. 209 H-J	3.7	Applegate and Espinosa-Arrubarrena[4]	400	Applegate and Espinosa-Arrubarrena[4]	597.22
	P	Cappetta[1]: fig. 209 M-N	1.6	Applegate and Espinosa-Arrubarrena[4]	400	Applegate and Espinosa-Arrubarrena[4]	597.22
O. angustidens	A	Gottfried and Fordyce[6]: fig 3A	10.4	Gottfried and Fordyce[6]	900	Gottfried and Fordyce[6]	7833.60
	L	Gottfried and Fordyce[6]: fig 3F	4.3	Gottfried and Fordyce[6]	900	Gottfried and Fordyce[6]	7833.60
	P	Gottfried and Fordyce[6]: fig 3I	1.9	Gottfried and Fordyce[6]	900	Gottfried and Fordyce[6]	7833.60
O. chubutensis	A	Perez et al.[3]: fig. 5	8.8	Perez et al.[3]	600	Perez et al.[3]	2162.96
	L	Perez et al.[3]: fig. 5	7.3	Perez et al.[3]	600	Perez et al.[3]	2162.96
	P	Perez et al.[3]: fig. 5	2.9	Perez et al.[3]	600	Perez et al.[3]	2162.96
O. megalodon	A	Gordon Hubbell collection (ds1022 UA1)	16.8	Applegate and Espinosa-Arrubarrena[4]	1500	Shimada[7]	39637.80
	L	Gordon Hubbell collection (ds1022 UL3)	15	Gordon Hubbell collection natural tooth set	1500	Shimada[7]	39637.80
	P	Gordon Hubbell collection (ds1022 UL8)	3.2	Gordon Hubbell collection natural tooth set	1500	Shimada[7]	39637.80

Supplementary Table 1. Details of planar models, tooth height, and body dimensions considered in the present study. In *O. chubutensis* and *O. megalodon*, lateral and posterior tooth heights were extrapolated according to the proportions in the tooth sets from Perez et al.[3] and Gordon Hubbell collection. For this, the largest tooth record reported in the literature was considered as the anterior tooth height in these species.

Taxon	*Tooth*	*Element size (mm)*	*Number of elements*	*Number of nodes*
O. obliquus	Anterior	0.30	33046	16869
O. obliquus	Lateral	0.30	29323	14984
O. obliquus	Posterior	0.10	40348	20527
O. auriculatus	Anterior	0.20	42787	21770
O. auriculatus	Lateral	0.20	42922	21834
O. auriculatus	Posterior	0.10	39775	20238
O. angustidens	Anterior	0.50	31453	16041
O. angustidens	Lateral	0.20	37739	19218
O. angustidens	Posterior	0.10	41985	21327
O. chubutensis	Anterior	0.40	31655	16111
O. chubutensis	Lateral	0.40	36730	18676
O. chubutensis	Posterior	0.20	32436	16507
O. megalodon	Anterior	0.90	33342	16950
O. megalodon	Lateral	0.80	36798	18689
O. megalodon	Posterior	0.25	30110	15312

Supplementary Table 2. Summary of mesh parameters.

Taxon	*Tooth*	*SA (m^2)*	*Puncture*		*Draw*		
			F (N) absolute	F (N) scaled	F (N)	Nodes in load	F (N) per node
O. obliquus	Anterior	1.43E-03	6988	5369	55	165	0.332
O. obliquus	Lateral	1.27E-03	10324	4759	49	126	0.385
O. obliquus	Posterior	1.95E-04	13661	732	7	127	0.059
O. auriculatus	Anterior	8.27E-04	2950	3103	32	175	0.181
O. auriculatus	Lateral	7.83E-04	4359	2937	30	133	0.225
O. auriculatus	Posterior	1.80E-04	5767	674	7	109	0.063
O. angustidens	Anterior	3.79E-03	16552	14219	145	161	0.900
O. angustidens	Lateral	7.27E-04	24456	2726	28	149	0.187
O. angustidens	Posterior	2.02E-04	32360	757	8	158	0.049
O. chubutensis	Anterior	3.76E-03	6988	14099	144	146	0.984
O. chubutensis	Lateral	2.83E-03	10324	10625	108	149	0.727
O. chubutensis	Posterior	6.23E-04	13661	2339	24	110	0.217
O. megalodon	Anterior	1.31E-02	49051	49051	500	162	3.086
O. megalodon	Lateral	1.15E-02	72481	43207	440	164	2.686
O. megalodon	Posterior	9.11E-04	95911	3418	35	98	0.356

Supplementary Table 3. Finite element model characteristics and loading scenarios. *F* force, *SA* surface area.

Supplementary references

1. Cappetta, H. Chondrichthyes–Mesozoic and Cenozoic Elasmobranchii: Teeth (Verlag F, Pfeil, 2012).
2. Diedrich, C. White and megatooth shark evolution and predation origin onto seals, sirenians and whales. *Nat. Sci.* **5**, 1203-1218 (2013).
3. Perez, V. J., Godfrey, S. J., Kent, B. W., Weems, R. E. & Nance, J. R. The transition between *Carcharocles chubutensis* and *Carcharocles megalodon* (Otodontidae, Chondrichthyes): lateral cusplet loss through time. *J. Vertebr. Paleontol.* **38**, e1546732 (2018).
4. Applegate, S. P. & Espinosa-Arrubarrena, L. The fossil history of *Carcharodon* and its possible ancestor, *Cretolamnia*: A study in tooth identification. In *Great White Sharks: The Biology of Carcharodon carcharias, Ch 4* (eds Klimley, A. P. & Ainley, D. G.) (Academic Press, San Diego, 1996).
5. Biton-Porsmoguer, S. Posible área de reproducción de *Otodus obliquus* (Lamniformes: Lamnidae) del Paleoceno en la cuenca de Ganntour (Marruecos). *UMC* hal-01660273 (2017).
6. Gottfried, M. D. & Fordyce, R. E. An associated specimen of *Carcharodon angustidens* (Chondrichthyes, Lamnidae) from the Late Oligocene of New Zealand, with comments on *Carcharodon* interrelationships. *J. Vertebr. Paleontol.* **21**, 730-739 (2001).
7. Shimada, K. The size of the megatooth shark, *Otodus megalodon* (Lamniformes: Otodontidae), revisited. *Hist. Biol.* 1-8 (2019).
